# Intranasal delivery of 9-cis retinoic acid reduces beta-amyloid deposition via inhibiting astrocyte-mediated inflammation

**DOI:** 10.18632/aging.102970

**Published:** 2020-03-25

**Authors:** Hong Zhao, Shuo Li, Zhuo Li, Shuo Yang, Dandan Li, Jiaolin Zheng, Hongmei Gao, Ling Yun, YingLi Gu, Longxuan Li, Jing Zhao, Yuan Fu

**Affiliations:** 1 Department of Neurology, The Fourth Hospital of Harbin Medical University, Harbin, China; 2 Department of Ultrasonography, The Fourth Hospital of Harbin Medical University, Harbin, China; 3 Department of Endocrinology, The First Hospital of Jilin University, Changchun, China; 4 Department of Neurology, The Second Hospital of Heilongjiang Province, Harbin, China; 5 Department of Neurology, The Second Hospital of Harbin Medical University, Harbin, China; 6 Department of Neurology, Gongli Hospital of The Second Military Medical University, Shanghai, China; 7 Department of Neuroscience, Mayo Clinic, Jacksonville, FL 32224, USA

**Keywords:** Alzheimer's disease, beta-amyloid, 9-cis retinoic acid, intranasal delivery, astrocyte activation

## Abstract

Alzheimer's disease (AD) is associated with the accumulation and deposition of a beta-amyloid (Αβ) peptide in the brain, resulting in increased neuroinflammation and synaptic dysfunction. Intranasal delivery of targeted drugs to the brain represents a noninvasive pathway that bypasses the blood-brain barrier and minimizes systemic exposure. The aim of this study was to evaluate the therapeutic effect of intranasally delivered 9-cis retinoic acid (RA) on the neuropathology of an AD mouse model. Herein, we observed dramatically decreased Αβ deposition in the brains of amyloid precursor protein (APP) and presenilin 1 (PS1) double-transgenic mice (APP/PS1) treated intranasally with 9-cis RA for 4 weeks compared to that in the brains of vehicle-treated mice. Importantly, intranasal delivery of 9-cis RA suppressed Αβ-associated astrocyte activation and neuroinflammation and ultimately restored synaptic deficits in APP/PS1 transgenic mice. These results support the critical roles of Αβ-associated neuroinflammation responses to synaptic deficits, particularly during the deposition of Αβ. Our findings provide strong evidence that intranasally delivered 9-cis RA attenuates neuronal dysfunction in an AD mouse model and is a promising therapeutic strategy for the prevention and treatment of AD.

## INTRODUCTION

Alzheimer's disease (AD), the most prevalent neurodegenerative disorder, is characterized by the presence of extracellular amyloid plaques composed of amyloid-β (Aβ) and intracellular neurofibrillary tangles [1, 2]. Mounting evidence indicates that Aβ accumulation and aggregation are associated with a toxic cascade triggered by neuroinflammation, which further results in synaptic loss and cognitive dysfunction [3]. The levels of Aβ in the brain are regulated by an innate immune response [4, 5], and Aβ activates an inflammatory response that ultimately drives its uptake and clearance from astrocytes and microglia in the brain [6]. Because astrocytes are key regulators of the brain’s inflammatory response [7], elucidating the mechanisms by which Αβ initiates this inflammatory cascade is crucial to understand the interplay between astrocytes and neuronal viability in AD.

9-cis Retinoic acid (RA), a biologically active derivative of vitamin A, has been shown to control a wide range of biological processes, including cell proliferation, differentiation, and morphogenesis [8]. It is well known that 9-cis RA regulates the activity of target cells via their nuclear receptors RA receptors (RARs) and retinoid X receptors (RXRs) [9, 10]. In vitro studies have indicated that 9-cis RA exerts immunomodulatory and anti-inflammatory effects on various cell types [8, 11, 12]. Mice that carry mutated versions of RAR and/or RXRs also show deficits in spatial learning and memory [13]. The impairment of spatial learning and memory and depression of synaptic plasticity that occur in vitamin A-deprived rodents also occur as rodents age [14]. Importantly, clinical evidence has shown defective retinoid transport and functions in the AD brain, suggesting that increasing the availability of RA in the brain may prevent or decrease Aβ-associated neurodegeneration [15]. However, to date, no conclusive experimental evidence obtained from AD animal models shows a therapeutic effect of 9-cis RA on AD.

In the present study, we examined the effects of intranasally delivered 9-cis RA on amyloid precursor protein (*APP*) and presenilin 1 (*PS1*) double-transgenic mice (*APP/PS1*). Six-month-old *APP/PS1* mice were intranasally treated with 20 μg of 9-cis RA for 4 weeks, which effectively reduced the Αβ burden. More intriguingly, 9-cis RA treatment significantly alleviated astroglial activation and synaptic loss in the brains of *APP/PS1* mice.

## RESULTS

### 9-cis RA reduces Aβ deposition in an amyloid mouse model

**Figure 1:**
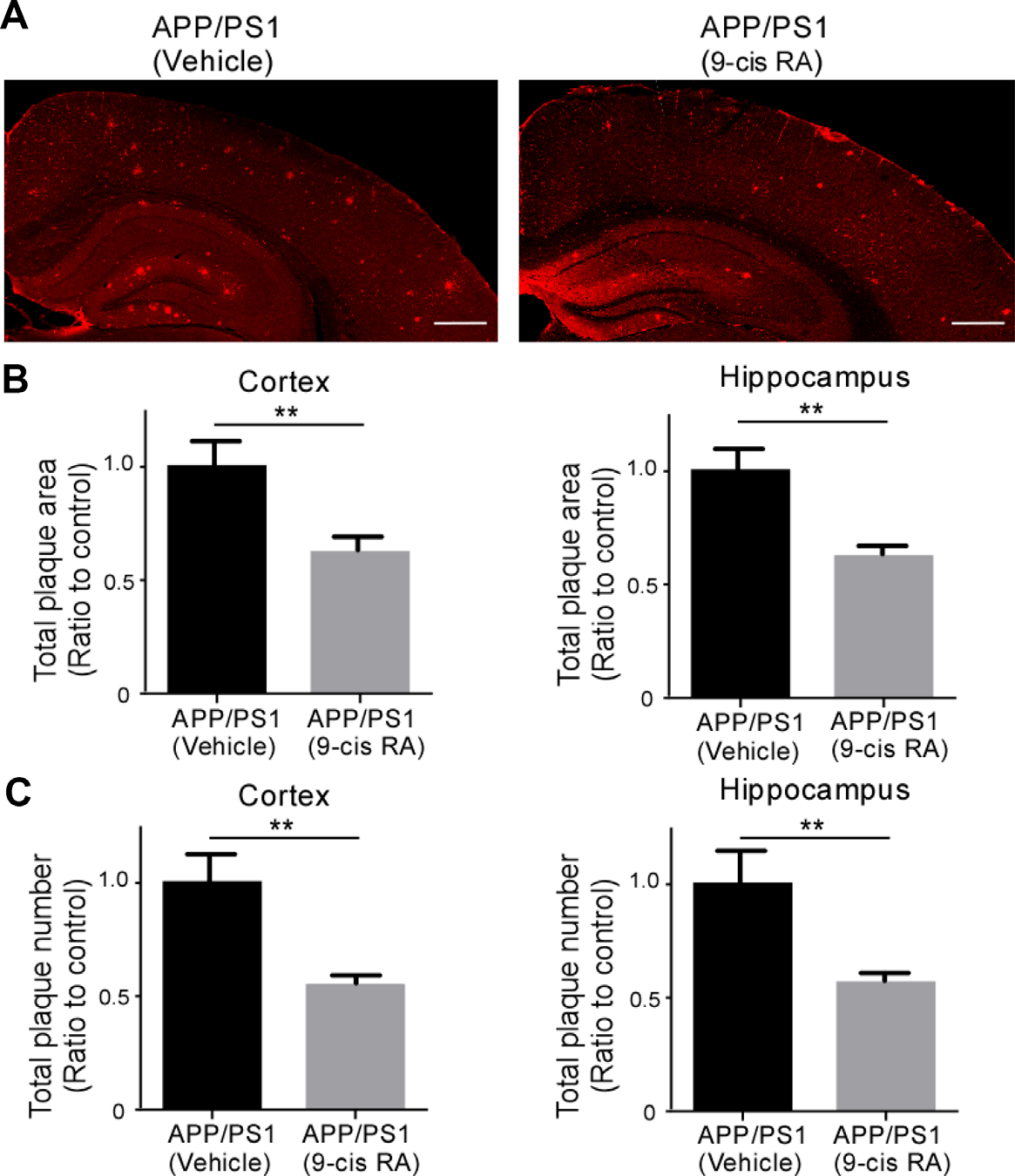
**Treatment with 9-cis RA reduced the level of Αβ deposition in APP/PS1 mice compared with vehicle-treated control mice.** (**A**) Representative images of Αβ staining in the frontal cortex and hippocampus of APP/PS1 mice treated with vehicle as a control (left) or 9-cis RA (right). Scale bars, 500 μm. (**B**) Stereological quantification of the Αβ volume in the cortex (left) and hippocampus (right). (**C**) Stereological quantification of the Αβ numbers in the cortex (left) and hippocampus (right). Values from multiple images of each section that cover most of the region of study were averaged per animal per experiment. Data represent the mean ± SEM (n=6). **, p<0.01.

To determine whether intranasal delivery of 9-cis RA affects amyloid pathology, we treated 6-month-old *APP/PS1* mice for a period of 4 weeks, as these mice are known to develop amyloid plaques at 5 to 6 months [16]. The brain sections were immunostained with an anti-Aβ antibody (82E1), and the extent of Aβ deposition in the cortical and hippocampal brain regions of *APP/PS1* mice was captured by confocal microscopy (Figure 1A). Treatment of *APP/PS1* mice with 9-cis RA significantly reduced the plaque burden in the cortical and hippocampal regions. Immunohistochemical analysis of 82E1 immunoreactive plaques in cortical slices from *APP/PS1* mice revealed an ~40% reduction in the total areas of Aβ deposits compared with those in cortical slices from their vehicle-treated *APP/PS1* littermates (Figure 1B). More importantly, the number of total plaques was also reduced in the cortex and hippocampus of 9-cis RA-treated *APP/PS1* mice by ~45% and ~43%, respectively (Figure 1C). These data demonstrate that 9-cis RA reduces the levels of Aβ deposition in the brains of *APP/PS1* mice.

### 9-cis RA decreases amyloid-associated neuroinflammation in an amyloid mouse model

**Figure 2:**
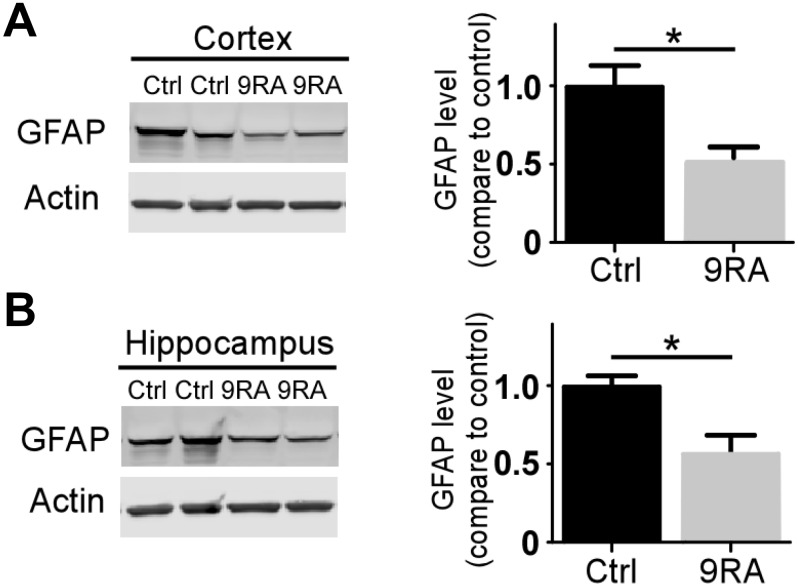
**9-cis RA reduced Αβ-associated gliosis.** (**A**, **B**) The levels of GFAP in the cortex (n=4/group) and hippocampus (n=4/group) were examined by western blotting. Data represent the mean ± SEM. *, p<0.05.

**Figure 3:**
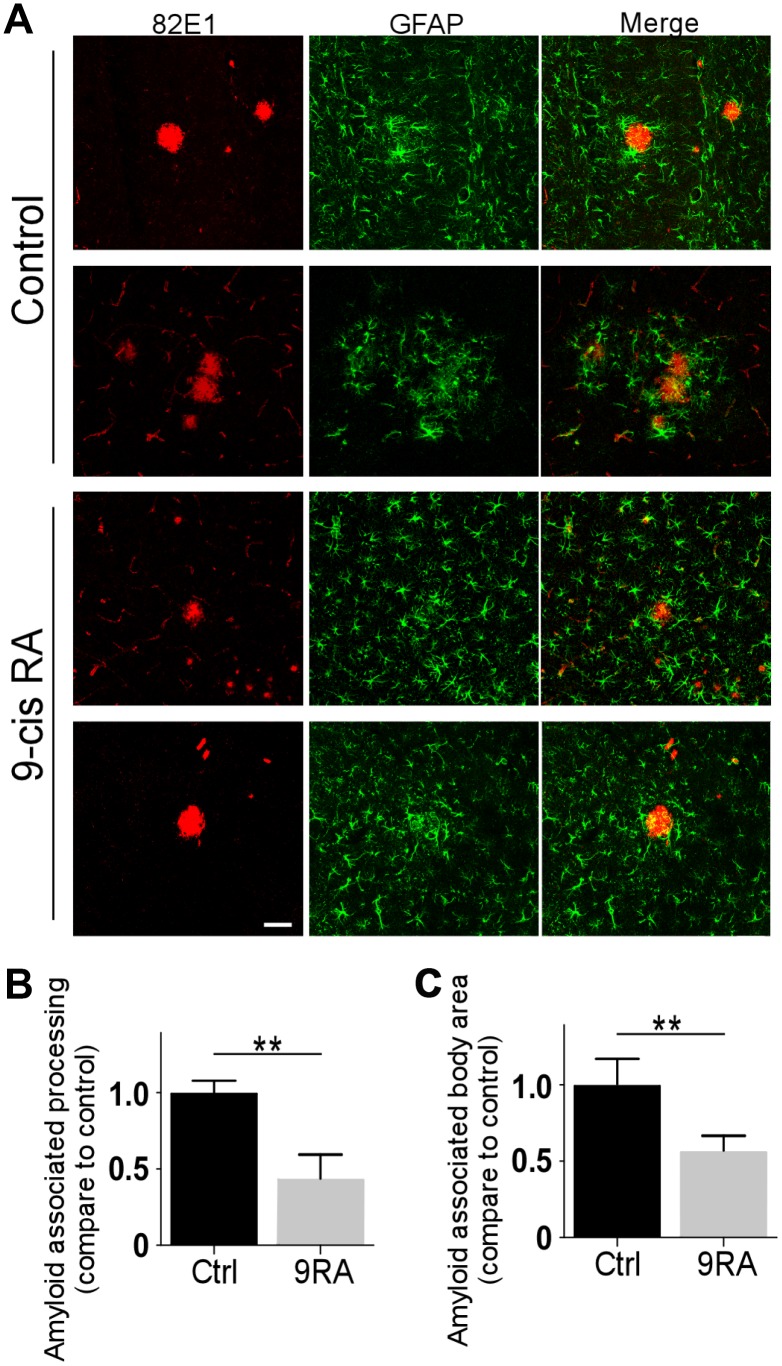
**9-cis RA suppressed the activation of astrocytes in APP/PS1 mice.** (**A**) Representative images of GFAP and 82E1 immunochemistry in coronal sections from 7-month-old APP/PS1 animals treated with 9-cis RA (bottom) or vehicle (upper). (**B**) Quantification of amyloid-associated astrocyte processes compared with the Ctrl (vehicle). (**C**) Quantification of amyloid-associated astrocyte bodies compared with the Ctrl (vehicle). Scale bars, 50 μm. Data represent the mean ± SEM (n=6). **, p<0.01.

Abnormal activation of astrocytes has been observed in the brains of AD patients and *APP* transgenic mouse models [17, 18]. To determine the extent of Aβ-mediated astrogliosis upon 9-cis RA treatment, we examined GFAP-positive reactive astrocytes by immunofluorescence staining and western blot. Interestingly, 9-cis RA-treated *APP/PS1* mice displayed significantly fewer GFAP-positive astrocytes in the cortex (Figure 2A) and hippocampus (Figure 2B) than the vehicle-treated mice, indicating that 9-cis RA had an anti-inflammatory effect on Aβ-mediated neuroinflammation. Next, we evaluated astroglial reactivity surrounding amyloid deposits in both 9-cis RA- and vehicle-treated *APP/PS1* animals. Treatment with 9-cis RA significantly reduced the levels of GFAP-immunoreactive astrocytes surrounding amyloid plaques in transgenic animals at 7 months old, as determined by immunostaining (Figure 3A). Quantitative analysis of amyloid-associated astrocyte processes (Figure 3B) and body area (Figure 3C) revealed that 9-cis RA treatment dramatically reduced the level of reactive astrocytes associated with Aβ-positive plaques in the brains of 7-month-old APP/PS1 mice. These data suggest that 9-cis RA treatment can reduce amyloid deposition through its anti-inflammatory function.

### Treatment with 9-cis RA rescued synaptic integrity in an amyloid mouse model

**Figure 4:**
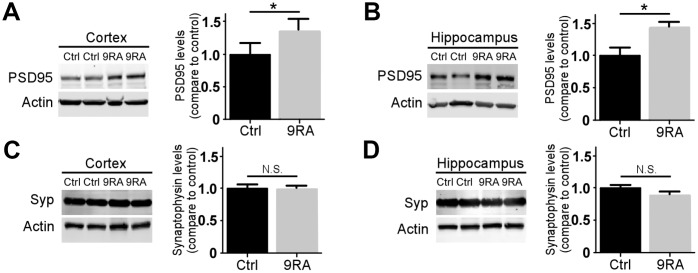
**9-cis RA rescued amyloid-associated synaptic loss.** The levels of the postsynaptic marker PSD-95 (**A**, **B**) and presynaptic marker synaptophysin (Syp) (**C**, **D**) in the cortex (left) and hippocampus (right) were examined by western blotting. Data represent the mean ± SEM (n=4). *, p<0.05.

Synaptic loss occurs early during AD progression and is one of the first signs of the neurodegenerative process [19, 20]. To further understand how 9-cis RA affects synaptic changes associated with Aβ, we examined the levels of pre- and postsynaptic markers in the brains of *APP/PS1* mice. The level of postsynaptic density 95 (PSD-95) (Figure 4A, 4B), but not that of synaptophysin (Figure 4C, 4D), was significantly increased in the 9-cis RA-treated mice, suggesting that 9-cis RA might ameliorate Aβ-associated synaptic impairment.

### 9-cis RA decreased the levels of proinflammatory cytokines in an amyloid mouse model

**Figure 5:**
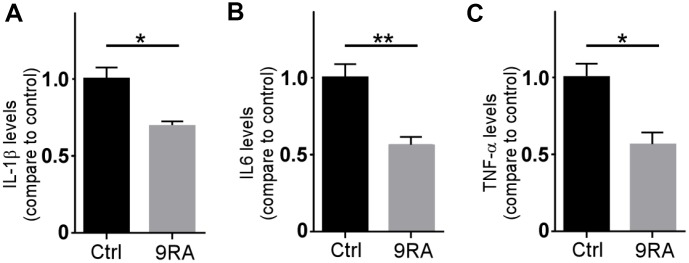
**9-cis RA reduced amyloid-associated neuroinflammation.** (**A**–**C**) The levels of IL-1β, IL-6, and TNF-α in the cortex of APP/PS1 were examined by ELISA. Data represent the mean ± SEM (n=6). *, P < 0.05.

Proinflammatory cytokines released by microglia are thought to have a central role in the AD neuroinflammation [21, 22]. Activated astrocytes serve as functional barriers and have the potential to release diverse molecules [23, 24]. To examine the effect of 9-cis RA on proinflammatory cytokines, we analyzed the levels of proinflammatory cytokines in the brain. We found that 9-cis RA decreased the levels of IL-6, IL-1β, and TNF-α compared with those in the vehicle control group (Figure 5A–5C). Consistent with the effects of 9-cis RA on amyloid deposition, the levels of proinflammatory cytokines were significantly reduced in the mice treated with 9-cis RA. These results indicate that 9-cis RA reduces the activation of astrocytes by decreasing proinflammatory cytokine expression.

## DISCUSSION

Excessive accumulation and aggregation of Aβ in senile plaques are the first events leading to AD-related dementia [6, 25]. Numerous pharmacological approaches and mechanical strategies to prevent and treat AD have targeted Aβ production, accumulation, and/or aggregation and have most recently focused on presymptomatic patients who are likely to develop AD [26, 27]. Neuroinflammation occurs in the brains of patients with symptomatic AD and has a critical impact on the neurodegenerative pathology of the disease [23]. Targeting Aβ-induced neuronal cell death has been demonstrated to play an important therapeutic role in modifying AD progression [28]. We previously found that 9-cis RA decreases cell-associated Aβ levels in astrocytes, which is beneficial for accelerating Aβ clearance [10]. In this study, we examined the effects of 9-cis RA delivered intranasally on AD pathogenesis in *APP/PS1* mice, as this route is a minimally invasive way to repeatedly deliver drugs to animals. Although the blood-brain barrier is impaired in AD, it can still prevent exogenous compounds from entering the brain parenchyma [29]. Small molecules administered intranasally can pass through the blood-brain barrier, avoid first-pass metabolism, and reduce nonselective effects in the periphery [30, 31]. Our findings indicated that 9-cis RA treatment, for as little as 4 weeks, inhibited and possibly reversed the accumulation of Aβ deposits in *APP/PS1* double-transgenic mice. The 9-cis RA-treated *APP/PS1* mice showed significantly decreased levels of activated astrocyte markers and proinflammatory cytokines and elevated levels of synaptic markers in the cortex and/or hippocampal regions compared with those in the vehicle-treated *APP/PS1* mice.

RA has been considered a regenerative molecule in peripheral organs, as it is generated in multiple forms (all-trans, 9-cis, and 13-cis) from retinol by two sequential reactions after uptake [32–34]. 9-cis RA mainly interacts with RXRs to regulate the transcription of several target genes by binding RA response elements (RAREs) in DNA to maintain lipid metabolism and glucose homeostasis [35]. Exogenous 9-cis RA has been shown to play multiple roles, such as increasing neurite outgrowth from cultured adult Lymnaea neurons and accelerating remyelination in the injured central nervous system (CNS) through the proliferation of immortalized hippocampal progenitor cells [36, 37]. An in vitro study showed that 9-cis RA inhibits lipopolysaccharide (LPS)-induced inflammatory responses in human adherent monocytes [11]. Taken together, these data suggest that 9-cis RA has neurotrophic properties and may be potentially useful for AD.

In our AD mouse model, reactive astrocytes were intimately associated with amyloid plaques. Astrocytes can secrete inflammatory cytokines and generate toxicity, thus damaging and killing bystander neurons [7]. GFAP expression is associated with activated astrocytes, representing the inflammatory state of the CNS [38, 39]. We herein found decreased astrocyte activation in 9-cis RA-treated mice compared with vehicle-treated mice, indicating that astrocytes contributed to the reduced Aβ load in the treated mice. Compared to those in the control mice, the Aβ deposits in 9-cis RA-treated mice were associated with substantially less processing of activated astrocytes. Furthermore, the astrocytes in the 9-cis RA-treated mice were smaller than those in the control mice. Together, these results suggest that the Aβ in the plaques exhibited altered aggregation kinetics. Finally, it should be noted that the number of Aβ deposits was also significantly reduced in the brains of 9-cis RA-treated mice compared with control mice, indicating that fewer new deposits were formed during the four weeks of treatment.

Aβ deposition triggers a neuroinflammatory state, which plays a significant role in the progression of AD [23, 40]. Inflammatory components related to AD neuroinflammation include brain cells such as microglia and astrocytes, the complement system, and cytokines and chemokines [5]. Proinflammatory cytokines, such as IL-1β, IL-6, and TNF-α, play an important role in the development of AD [41, 42]. An in vitro study demonstrated that 9-cis RA suppresses the production of specific proinflammatory cytokines from activated primary glial cells [8]. However, how 9-cis RA affects the levels of proinflammatory cytokines in an AD mouse model remains unclear. In this study, we found that 9-cis RA, as an anti-inflammatory drug, decreased the levels of IL-1β, IL-6, and TNF-α in *APP/PS1* mice, suggesting that it could relieve the activation of astrocytes by reducing proinflammatory cytokine levels.

In addition, neuroinflammatory components may contribute to synapse loss and dysfunction in AD. PSD-95 is the most abundant scaffolding protein in the excitatory postsynaptic density, and its expression has been proven to decrease with the progression of memory loss in AD [43]. Treatment with 9-cis RA significantly increased the PSD-95 levels in both the cortices and hippocampi of *APP/PS1* mice compared to the vehicle-treated controls, reflecting the restoration of postsynaptic damage.

Overall, the data reported herein represent the first preliminary experimental evidence of the therapeutic effect of intranasally delivered 9-cis RA on AD. Additionally, we have provided a mechanism by which 9-cis RA modulates Aβ-related pathology in a mouse model. We have shown a mechanistic linkage between reactive astrocytes and proinflammatory cytokines, which has not been previously documented in the brain, that facilitates amyloid deposition and accelerates synaptic loss. These data suggest that 9-cis RA, as an anti-inflammatory drug, represents a promising therapeutic approach for AD.

## MATERIALS AND METHODS

### Animals

All animal care protocols and procedures were performed in accordance with the Animal Scientific Procedures Act and were approved by Harbin Medical University. *APP*swe/PS1Δe9 (*APP/PS1*) transgenic mice [B6C3-Tg (*APP*swe, PSEN1dE9)85Bdo/J] were obtained from the Model Animal Research Center of Nanjing University, China [44]. Briefly, 20 μg of 9-cis RA (1 μg/μl, ab141023, Abcam) or vehicle (10% DMSO in saline) was administered to 6-month-old *APP/PS1* mice intranasally every 2 days for 4 weeks as previously described [45]. The animal brains were then harvested, and one hemisphere was fixed and processed for immunohistochemistry. The hippocampus and cortex were dissected from the other hemisphere, snap-frozen and stored at 80°C until protein extraction. Approximately equal numbers of male (n=16) and female (n=16) transgenic mice were used for all experiments.

### Immunohistochemistry

Postfixed hemispheres were sectioned (20 μm) on a cryostat and stored in PBS/glycerol (50:50) until use. Alternate sections were blocked with 5% BSA and stained with the appropriate primary antibody: anti-amyloid (82E1, 1:1000, Immunobiological Laboratories) and anti-GFAP (1:1000, Millipore). The sections were incubated with the appropriate Alexa Fluor-conjugated secondary antibodies, and images were captured on a confocal laser scanning fluorescence microscope (model LSM510 invert; Carl Zeiss, Germany).

### Western blot analysis

Protein concentrations in brain extracts were measured using the BCA method (Pierce), and equal amounts of protein from the homogenized lysates were loaded onto SDS-PAGE gels and transferred to PVDF membranes. After the membranes were blocked, proteins were detected with one of the following primary antibodies: anti-GFAP (1:500, Millipore), anti-PSD-95 (1:200, Cell Signaling Technology), or anti-synaptophysin (1:200, Millipore). The membranes were probed with LI-COR IRDye secondary antibodies, and proteins were detected using the Odyssey infrared imaging system (LI-COR).

### ELISA quantification

The concentrations of IL-1β, IL-6, and TNF-α were measured using commercial kits (Biolegend) according to the manufacturers’ instructions. Briefly, plates were coated with a capture antibody, incubated overnight at 4°C, washed, and blocked for 1 hour at room temperature. Standard and experimental samples were added to the plate for 2 hours at room temperature. After washing, the plates were incubated with a detection antibody for 1 hour and then washed and incubated with an Avidin-HRP solution for 30 minutes. After washing the plates, substrate solution and stop solution were added to each well, and the absorbance was read at 450 nm and 570 nm.

### Statistical analysis

All quantified data represent an average of samples. Statistical analyses were performed with Excel or GraphPad Prism software. Statistical significance was determined by either Student’s t-test or one-way analysis of variance (ANOVA) with Tukey’s post hoc test using GraphPad Prism 5. P < 0.05 was considered statistically significant. The levels of significance are indicated as follows: *P < 0.05, **P < 0.01.

### Data and materials availability

Data from these experiments are available from the corresponding author upon reasonable request.
